# Photoprotective pigment plasticity and cold acclimation strategies in *Cryptomeria japonica* across two common gardens

**DOI:** 10.48130/forres-0025-0015

**Published:** 2025-07-31

**Authors:** Qingmin Han, Norihisa Kusumoto, Seiichi Kanetani, Yoshihisa Suyama, Yuki Tsujii, Daisuke Kabeya, Yoshihiko Tsumura, Kentaro Uchiyama

**Affiliations:** 1 Department of Plant Ecology, Forestry and Forest Products Research Institute (FFPRI), 1 Matsunosato, Tsukuba, Ibaraki 305-8687, Japan; 2 Department of Forest Resource Chemistry, FFPRI, 1 Matsunosato, Tsukuba, Ibaraki 305-8687, Japan; 3 Kyushu Research Center, FFPRI, 4-11-16 Kurokami, Kumamoto 860-0862, Japan; 4 Field Science Center, Graduate School of Agricultural Science, Tohoku University, Osaki 989-6711, Japan; 5 Faculty of Life and Environmental Sciences, University of Tsukuba, Tsukuba, Ibaraki 305-8572, Japan; 6 Department of Forest Molecular Genetics and Biotechnology, 1 Matsunosato, Tsukuba, Ibaraki 305-8687, Japan

**Keywords:** Acclimation, Chlorophyll, Japanese cedar, Photosynthesis, Plasticity, Rhodoxanthin, Xanthophyll cycle, Winter stress

## Abstract

To cope with winter stress from low temperatures and excessive light, evergreen conifers employ seasonal adjustments in photosynthetic function. Understanding these regulatory mechanisms is critical for predicting coniferous forest responses to climate change and their role in the global carbon cycle. To assess variation in cold acclimation strategies, the following were analyzed: pigment composition, photochemical efficiency of photosystem II, and photosynthetic parameters in three to five provenances (Prv) of *Cryptomeria japonica* grown in two common gardens (CGs) with contrasting climates. All Prv exhibited winter chlorophyll reduction, increased xanthophyll cycle, lutein pigments, and rhodoxanthin accumulation, reflecting conserved photoprotective responses. However, needle chlorophyll concentrations were unexpectedly higher in the colder site, especially in the northernmost Prv, suggesting genotype-specific plasticity. Higher rhodoxanthin levels in the hotter sites indicated a trade-off between the xanthophyll cycle and rhodoxanthin-mediated protection governed by winter severity. Despite these differences, values of photochemical efficiency of photosystem II were similar among Prv within each garden, though consistently higher in the hotter garden. No significant variation in photosynthetic capacity was detected among the three Prv measured. This local adaptation is further supported by high phenotypic plasticity in pigment composition and leaf morphology. These findings highlight the diverse and flexible mechanisms by which *C. japonica* regulates pigment composition, enabling sustained photosynthesis across seasonal extremes, and suggest a role for both winter cold and summer heat in shaping local adaptation in this widely distributed conifer.

## Introduction

Evergreen conifers endure harsh winter conditions characterized by low temperatures and high light intensity. While cold temperatures inhibit enzymatic activity and photosynthetic carbon assimilation, they do not prevent light absorption, potentially leading to excessive excitation pressure in photosystem II (PSII) and consequent photooxidative damage to thylakoid membranes^[1]^. To mitigate such stress, conifers must either reduce light absorption or dissipate excess absorbed energy through photoprotective mechanisms.

An important photoprotective mechanism to winter conditions involves increased utilization of sustained forms of thermal energy dissipation: the conversion of light energy absorbed by chlorophyll (Chl) to heat^[2−4]^. During the growing season, dynamic engagement of the xanthophyll cycle (VAZ)—de-epoxidation of violaxanthin (V) to zeaxanthin (Z) via antheraxanthin (A)—is rapidly induced under excess light and reversed under low light conditions^[1,5−7]^. Seasonal acclimation involves modulation of the VAZ pool size^[8]^, allowing for efficient energy use while minimizing photodamage. Z accumulation is likely to occur in late autumn when conditions are still favorable for de-epoxidase activity^[1,3]^. Sustained Z accumulation facilitates transitioning from a 'light-harvesting' to a 'dissipating' state in PSII. Although winter increases in VAZ pool size are well-documented, their magnitude varies among species and environments^[3,4]^, and the regulatory mechanisms remain unresolved.

Cold acclimation also entails seasonal Chl reduction to limit light absorption^[3,9,10]^, though the degree and underlying mechanisms are unclear and species-dependent^[11]^. Some conifers even increase Chl in winter^[4]^, suggesting divergent strategies^[1,3,12]^. As Chl biosynthesis is slow, winter reduction may hinder spring photosynthesis recovery^[13−15]^. Moreover, structural rearrangements in the photosynthetic apparatus, such as altered thylakoid architecture in *Pinus sylvestris*^[1]^, contrast with mechanisms observed in co-occurring species like *Picea abies*^[12]^. Nevertheless, photosynthesis reactivates under favorable spring temperatures, as indicated by starch accumulation^[16,17]^. Coordinated seasonal variation in Chl and carotenoids is thus critical to maintaining energy balance, and intraspecific comparisons can yield novel insights.

In addition to the VAZ cycle, rhodoxanthin (Rho), a deep red ketocarotenoid, accumulates in gymnosperms such as *Cryptomeria*, *Metasequoia*, *Taxodium*, *Chamaecyparis,* and *Thuja*^[18−21]^. Rho biosynthesis originates from VAZ pigments^[22,23]^, and increases during the autumn-winter transition under stress^[14,21,24]^. Its role in light interception, localized near the leaf surface in chromoplasts, and its prevalence in sun-exposed leaves suggest a photoprotective function^[19,25]^. However, its physiological role and interactions with other pigments remain unclear.

**Figure 1:**
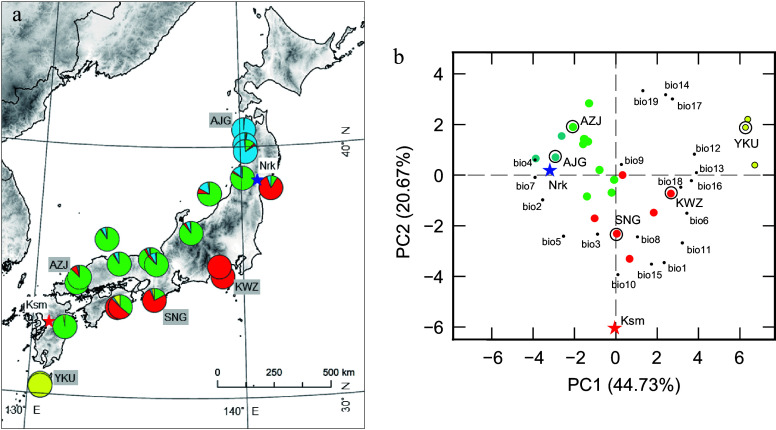
Original geographic distribution and principal component analysis (PCA) of climatic variables of *C. japonica* Prv. (a) Locations of the five Prv used in this study and the two CGs (CG, star). Colors represent genetic structure, modified from Uchiyama et al.^[33]^. (b) PCA of climatic variables in the Prv and CG. Prv and CG scores are represented by large circles and stars in the same colors as used in (a). Climatic variables are represented by small black circles. The climatic variables are annual mean temperature (bio1), mean diurnal range (bio2), isothermality (bio3), temperature seasonality (bio4), maximum temperature of the warmest month (bio5), minimum temperature of the coldest month (bio6), temperature annual range (bio7), mean temperature of the wettest quarter (bio8), mean temperature of the driest quarter (bio9), mean temperature of the warmest quarter (bio10), mean temperature of the coldest quarter (bio11), annual precipitation (bio12), precipitation of the wettest month (bio13), precipitation of the driest month (bio14), precipitation seasonality (bio15), precipitation of the wettest quarter (bio16), precipitation of the driest quarter (bio17), precipitation of the warmest quarter (bio18), and precipitation of the coldest quarter (bio19). Prv and CG abbreviations are listed in Table 1.

This study tests the hypothesis that intraspecific variation and plasticity in Chl, carotenoids, photosynthesis, and leaf morphology have co-evolved in Sugi (*C. japonica*), a widely distributed species with a recently developed genome assembly^[26]^. Natural populations form two genetic clusters (Fig. 1): 'Omote-sugi' (Pacific Ocean side) and 'Ura-sugi' (Sea of Japan side), which experience contrasting winter climates^[27]^. While prior studies have documented population-level variation in roots^[28]^, volatiles^[29,30]^, and growth-related traits^[31,32]^, pigment variation remains unexplored. To investigate cold acclimation strategies, pigment composition and photochemical efficiency of PSII (F_v_/F_m_) were measured in five provenances grown in two common gardens (CGs) because sustained A and Z levels have previously been associated with reductions in F_v_/F_m_^[4]^. The light response curves of photosynthesis in three Prv were also measured to assess variation in photosynthetic parameters. The following hypotheses were tested: (1) To cope with the harsher winter, populations from colder regions exhibit lower Chl and higher VAZ and/or Rho concentrations; (2) Individuals grown in the warmer CGs have higher Chl and lower photoprotective pigments because of lower excitation pressure; (3) Lower F_v_/F_m_ values and reduced photosynthetic capacity are observed in both colder CGs and populations from colder regions.

## Materials and methods

### Site description and plant material

Provenance trials of Japanese cedar (*C. japonica*) were established in Miyagi Prefecture (Nrk) in 2014 and in Kumamoto Prefecture (Ksm) in 2017 by planting two-year-old cuttings from 23 Prv (Fig. 1). Each Prv was represented by five to ten clones, with three ramets per clone, arranged in a randomized complete block design. Each CG comprised approximately 1,000 trees. Three years after the establishment of the CG, the survival rate was 86.4% and 68.9% in Nrk and Ksm, respectively. For this study, five representative Prv were selected based on genetic clustering and the environmental characteristics of their original habitats (Fig. 1a). The selected Prv have notable differences in annual precipitation (1,709.6−4,722.3 mm), minimum temperature of coldest month (−1.2 to −10.8 °C), and temperature annual range (28.3−41.6 °C) (Table 1). Detailed information on the Prv and the two CG sites is provided in Table 1. Monthly rainfall, sunshine hours, daily mean temperature, and daily minimum and maximum temperature in the years when leaves were sampled are presented in Supplementary Fig. S1. Individual tree height in the winter when leaves were sampled ranged from 139 to 422 cm in 2019 at Nrk, and from 62 to 228 cm in 2020 at Ksm.

**Table 1:** General data on Prv and CGs used in this study.

Prv (abbreviation)	Latitude	Longitude	Altitude (m)	AMT (°C)	AP (mm)	MTCM (°C)	MTWQ (°C)	TAR (°C)	PWM (mm)	PCQ (mm)
Ajigasawa, Aomori (AJG)	40°41'	140°12'	280	11.1	1,709.6	−9.0	22.1	41.6	204.7	465.9
Azouji, Shimane (AZJ)	34°29'	131°59'	1,041	9.4	2,539.1	−10.8	19.9	40.4	301.6	432.3
Kawazu, Shizuoka (KWZ)	34°50'	139°00'	620	14.7	3,645.9	−2.9	23.3	33.5	441.4	455.2
Shingu, Wakayama (SNG)	33°53'	135°43'	560	13.9	3,105.8	−6.2	24.1	39.8	448.2	317.5
Yakushima, Kagoshima (YKU)	30°19'	130°34'	1,025	13.8	4,722.3	−1.2	21.3	28.3	835.0	699.9
Common gardens (CG)										
Kumamoto trial site (Ksm)	32°42'	130°45'	30	17.9	1,908.8	−2.5	27.7	38.9	436.1	191.3
Miyagi trial site (Nrk)	38°46'	140°45'	290	11.4	1,684.0	−9.3	22.2	42.6	181.6	355.2
Averaged values between 1981 and 2000 from www.worldclim.org/data/bioclim.html#google_vignette. AMT: annual mean temperature (bio1), AP: annual precipitation (bio12), MTCM: minimum temperature of coldest month (bio6), TAR: temperature annual range (bio7), MTWQ: mean temperature of warmest quarter (bio10), PWM: precipitation of wettest month (bio13), PCQ: precipitation of coldest quarter (bio19).										

### Leaf sampling

Leaves were sampled from three trees across three to five clones of each Prv during summer and winter at each CG: 30 July 2019 and 6 February 2020 at Nrk, and 5 August 2020 and 2 February 2021 at Ksm, respectively. Collections were made twice per season—at noon and 2 h after sunset on clear days. From each tree, two mature current-year needles (~100 mg fresh weight) were harvested from sun-exposed upper crown positions. One sample was immediately flash-frozen in liquid nitrogen in the field and stored at −80 °C for pigment analysis. These samples were lyophilized for 48 h, and their dry mass was measured under dim green light. The second sample was kept in a cool box for leaf area measurement. Projected leaf areas were measured using a scanner (GT-X900, EPSON, Tokyo, Japan) and LIA32 software (www.agr.nagoya-u.ac.jp/~shinkan/LIA32/index-e.html). Samples were then dried at 70 °C for 48 h, and dry mass was recorded to calculate leaf mass per area (LMA).

### Pigment analysis

Pigments in each leaf were extracted with acetone^[34]^, and quantified with an HPLC system (Agilent 1200, Agilent Technologies Inc., CA, USA) following the method of Gilmore & Yamamoto^[35]^ with modifications by Han et al.^[19]^. In preliminary tests, pigment composition quantified from lyophilized tissues did not differ from that of liquid nitrogen–frozen tissues (data not shown). Briefly, pigments from each lyophilized sample (< 70 mg) were extracted three times with 500 μL of 100% acetone. The pooled extracts were centrifuged, and 200 μL of the supernatant was filtered through 0.2 μm Mini-Uni Prep™ filters (Whatman, Cytiva, IN, USA). Pigments were separated using a Wakosil-II 5C18 column (4.6 mm × 250 mm; Fujifilm Wako, Osaka, Japan) at 30 °C and a flow rate of 1.0 mL·min^−1^. Elution began with a mixture of acetonitrile, methanol, and 100 mM Tris-HCl (75:12:4, v/v, pH 8.0) for 10 min, followed by a 10-min linear gradient to methanol : ethyl acetate (68:32, v/v), continued isocratically for 4 min. The column was re-equilibrated with the initial mobile phase for 10 min between samples. Pigment measured included chlorophyll *a* (Chl*a*) and Chl*b*, neoxanthin (Neo), lutein, V, A, Z, Rho, *α*-carotene (*α*-Car), and *β*-carotene (*β*-Car). Pigment concentrations were determined from calibration curves using authentic standards; all were purchased from CaroteNature (Münsingen, Switzerland) except Chl*a* and Chl*b* from Sigma-Aldrich (St. Louis, MO, USA). Total Chl was the sum of Chl*a* and Chl*b*.

### Photosynthesis and chlorophyll fluorescence measurements

Photochemical efficiency of photosystem II (F_v_/F_m_) was measured *in situ* using a Mini-Pam fluorometer (Walz, Effeltrich, Germany) after at least 2 h dark adaptation following sunset^[36]^. The same leaves were subsequently used for pigment analysis. Light-response curves of photosynthesis were measured in detached branches with a LI-6400 system (Li-Cor, Lincoln, NE, USA) under ten irradiance levels (1,600 to 0 μmol·m^−2^·s^−1^) at controlled temperatures (25 °C in winter, 28 °C in summer), relative humidity (VPD < 1.2 kPa) and CO_2_ concentration (400 ± 1.0 µmol·mol^−1^). From these curves, the light-saturated photosynthetic rate (A_max_) was calculated, alongside the apparent quantum yield of CO_2_ fixation (Ø), dark respiration rate (R_d_), and curve convexity (*θ*), using a non-rectangular hyperbola model^[37]^.

### Phenotypic plasticity

To assess phenotypic plasticity in response to environmental variation, the plasticity index (PIv) for LMA, pigments, and F_v_/F_m_ was calculated following Valladares et al.^[38]^. PIv for each trait was calculated using average values across all clones within each Prv at each CG:

$ \mathrm{P}\mathrm{I}\mathrm{v}_{\left(\mathrm{t}\mathrm{r}\mathrm{a}\mathrm{i}\mathrm{t}\right)}=\dfrac{\mathrm{M}\mathrm{a}\mathrm{x}\mathrm{i}\mathrm{m}\mathrm{u}\mathrm{m}\; \mathrm{v}\mathrm{a}\mathrm{l}\mathrm{u}\mathrm{e}-\mathrm{ }\mathrm{M}\mathrm{i}\mathrm{n}\mathrm{i}\mathrm{m}\mathrm{u}\mathrm{m}\; \mathrm{v}\mathrm{a}\mathrm{l}\mathrm{u}\mathrm{e}}{\mathrm{M}\mathrm{a}\mathrm{x}\mathrm{i}\mathrm{m}\mathrm{u}\mathrm{m}\; \mathrm{v}\mathrm{a}\mathrm{l}\mathrm{u}\mathrm{e}} $

PIv for pigments was the mean of all measured pigment components, including Chl*a*, Chl*b*, VAZ, Neo, lutein, *α*-Car, and *β*-Car in summer, and the surplus Rho in winter. Since pigment data were collected from multiple clones per Prv, PIv_(pigments)_ were also calculated by clone for each Prv and CG. PIv values range from 0 (no plasticity) to 1 (maximum plasticity).

### Statistical analysis

Given well-documented seasonal trends—VAZ pigments increasing and Chl decreasing in winter^[2−4,9,10]^—the effects of genotype (Prv) and environment (CG) on pigments, LMA, and F_v_/F_m_ were analyzed separately for each season using linear mixed-effects models (LMMs). Clone was treated as a random effect (random intercept). The carotenoid biosynthetic pathway divides from lycopene into *α*- and *β*-Car^[39]^, which are referred to as path A and path B, respectively. Lutein is produced from *α*-Car in path A and zeaxanthin from *β*-Car in path B. Further analyses were carried out on the seasonal variation in carotenoids of path A (sum of *α*-Car and lutein) and path B (sum of *β*-Car, VAZ, Rho, and Neo) using LMMs. Two-way ANOVA was employed to evaluate the effects of Prv and CG on photosynthetic parameters and the mean PIv of pigments. PCA was used to summarize variation in climatic variables and pigment composition across Prv and CG in each season. Nineteen bioclimatic variables for the period of 1981−2000 were extracted from WorldClim 2^[40]^. All statistical analyses were performed using R version 4.4^[41]^.

## Results

### Climatic variables

The first two PCs of the PCA on population-level climatic variation accounted for 63.40% of the total variance (Fig. 1b). PC1 explained 44.73% of the variance and was positively associated with precipitation of the wettest month and annual precipitation, while negatively associated with temperature annual range and temperature seasonality. The southernmost and northernmost populations were positioned at opposite ends of this axis. The Nrk CG was located at the negative end of PC1, reflecting a climate characterized by high temperature seasonality. PC2 accounted for 20.67% of the variance and was positively associated with precipitation during the coldest quarter and the driest month, but negatively associated with the mean temperature of the warmest quarter and precipitation seasonality. The Krm CG occupied the far negative end of PC2, indicative of a climate with a hot summer and pronounced precipitation seasonality.

### Pigment composition

The effects of Prv and CG on pigment concentrations varied depending on whether values were expressed per unit dry mass (Supplementary Table S1) or projected leaf area, due to differences in LMA (Supplementary Fig. S2). Because light absorption occurs at the leaf surface, comparisons were primarily made based on projected area.

**Figure 2:**
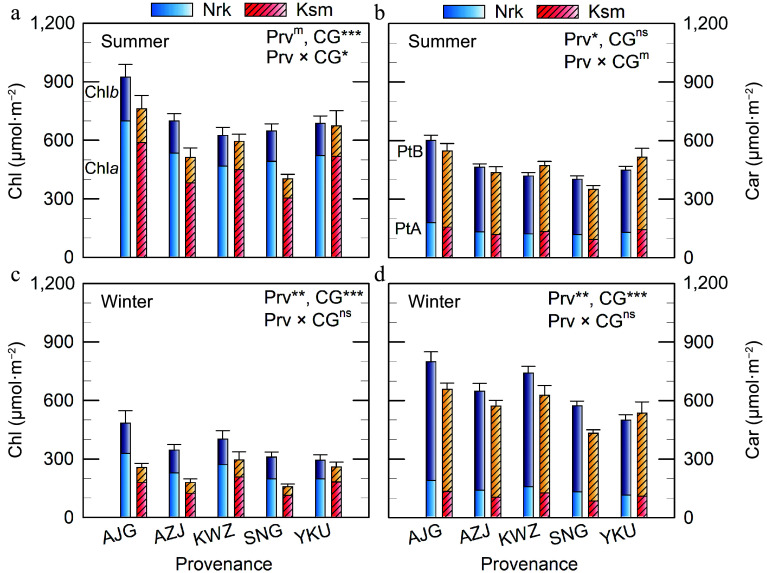
Total chlorophyll (Chl = Chl*a* + Chl*b*) and total carotenoid (Car) concentrations in needles of *C. japonica* from five Prv grown in two CGs located in Nrk and Ksm. Values represent means from three to five trees. Error bars indicate (a), (c) total Chl and (b), (d) total Car. Car of path A (PtA) is *α*-carotene + lutein, and Car of path B (PtB) is *β*-carotene + xanthophyll cycle (V + A + Z) + Rho + Neo*.* Prv abbreviations are listed in Table 1. Significant effects of Prv, CG, and their interaction are denoted as ^m^ *p* < 0.10, * *p* < 0.05, ** *p* < 0.01, *** *p* < 0.001, ^ns^ not significant.

In contrast to the initial hypothesis, needle Chl*a*, Chl*b,* and total Chl concentrations were higher in the colder CG than in the warmer CG (Fig. 2). A significant interaction between Prv and CG was observed in summer, but not in winter, indicating that genotype mediates plastic responses to the environment. Notably, the northernmost Prv (AJG) exhibited the highest Chl concentration, particularly when grown in the colder CG. In winter, Chl*b* levels declined to 79% and 48% of their summer values in the colder and warmer CG, respectively, resulting in a lower Chl*a*:*b* ratio in the colder CG (Supplementary Table S2). In contrast, the Chl*a*:*b* ratio was consistent across all Prv in summer.

Carotenoid of path A had less seasonal variation (*p* = 0.189), whereas that of path B increased in winter (Fig. 2, Table 2, *p* < 0.001). Significant differences in Car of path A, path B, and total Car were found between Prv in both seasons, and between CGs in winter, but not in summer.

**Table 2:** Pigment concentration (μmol·m^−2^) in needles of *C. japonica* originating from five Prv growing in two CGs and statistical summary.

Season	Prv	CG	*α*-carotene	Lutein	Path A	*β*-carotene	Neo	Path B	Chl*a*	Chl*b*
Summer	AJG	Ksm	25.6 ± 2.4	130.9 ± 8.9	155.83 ± 10.95	348.7 ± 21.7	25.7 ± 2.3	391.25 ± 25.97	588.3 ± 55.3	174.1 ± 13.0
		Nrk	43.7 ± 2.5	135.3 ± 6.4	178.89 ± 8.78	373.7 ± 15.7	34.4 ± 2.2	421.67 ± 18.93	700.0 ± 53.3	224.3 ± 12.1
	AZJ	Ksm	20.4 ± 1.9	97.8 ± 6.8	118.00 ± 8.57	286.7 ± 19.3	18.9 ± 1.4	318.00 ± 21.47	380.6 ± 38.4	131.8 ± 10.8
		Nrk	31.0 ± 1.7	100.9 ± 3.1	131.67 ± 4.80	295.4 ± 9.1	25.6 ± 0.9	332.22 ± 11.10	533.7 ± 32.6	165.9 ± 6.2
	KWZ	Ksm	22.1 ± 1.9	109.2 ± 4.9	132.22 ± 6.84	302.4 ± 13.8	20.4 ± 1.5	339.44 ± 15.91	449.4 ± 29.4	144.9 ± 8.0
		Nrk	29.9 ± 2.0	90.2 ± 3.5	120.56 ± 5.57	265.9 ± 10.0	23.1 ± 1.3	297.78 ± 11.90	467.9 ± 34.8	157.0 ± 7.7
	SNG	Ksm	13.5 ± 1.2	80.1 ± 4.1	93.18 ± 5.11	230.3 ± 10.9	14.0 ± 0.9	256.82 ± 13.17	304.0 ± 19.4	98.6 ± 5.2
		Nrk	29.6 ± 2.5	89.5 ± 3.8	118.33 ± 6.28	251.2 ± 10.2	22.6 ± 1.4	282.78 ± 12.12	491.7 ± 28.4	156.2 ± 9.1
	YKU	Ksm	24.6 ± 4.2	119.3 ± 10.2	143.33 ± 14.37	328.8 ± 25.4	21.7 ± 2.9	371.67 ± 31.98	518.2 ± 62.9	156.4 ± 16.5
		Nrk	32.2 ± 3.0	97.4 ± 4.7	129.38 ± 7.82	286.5 ± 10.7	23.3 ± 1.5	318.75 ± 12.68	522.0 ± 30.6	164.2 ± 9.9
	Statistics (*F* value)									
	Prv		2.94*	3.71**	3.76*	4.11*	2.84^m^	3.64*	2.15^ns^	2.93*
	CG		69.48***	0.20^ns^	4.17*	0.49^ns^	35.23***	0.61^n^	18.09***	36.49***
	Prv × CG		1.23^ns^	3.18*	2.20^m^	2.47*	1.51^ns^	2.46*	2.87*	2.07^m^
Winter	AJG	Ksm	0.7 ± 0.4	131.1 ± 6.9	132.27 ± 7.05	312.6 ± 26.4	16.6 ± 2.4	523.64 ± 29.27	177.8 ± 17.6	77.5 ± 5.5
		Nrk	4.2 ± 0.7	185.1 ± 9.0	189.44 ± 9.98	397.1 ± 42.2	28.5 ± 2.7	608.89 ± 44.01	326.1 ± 48.1	156.3 ± 16.2
	AZJ	Ksm	0.0 ± 0.0	104.1 ± 5.0	103.50 ± 4.99	254.1 ± 18.4	10.1 ± 0.9	467.00 ± 24.89	121.9 ± 14.1	56.0 ± 5.8
		Nrk	1.9 ± 0.5	136.2 ± 6.1	137.65 ± 6.33	292.5 ± 23.7	23.5 ± 1.8	509.41 ± 33.34	227.4 ± 20.7	116.4 ± 10.6
	KWZ	Ksm	0.0 ± 0.0	122.9 ± 9.2	124.71 ± 9.32	305.4 ± 31.0	14.5 ± 1.7	501.76 ± 43.46	207.1 ± 31.5	86.6 ± 10.2
		Nrk	3.2 ± 0.8	153.8 ± 7.4	156.67 ± 8.12	363.9 ± 25.7	25.7 ± 2.0	583.33 ± 25.46	268.9 ± 30.5	132.6 ± 13.4
	SNG	Ksm	0.0 ± 0.0	83.8 ± 4.6	83.18 ± 4.62	191.1 ± 13.0	9.8 ± 0.9	347.73 ± 15.62	111.4 ± 10.7	44.5 ± 4.4
		Nrk	1.9 ± 0.5	128.9 ± 5.6	130.56 ± 5.91	277.8 ± 17.1	21.2 ± 1.7	442.22 ± 18.09	196.7 ± 19.0	111.8 ± 9.1
	YKU	Ksm	0.0 ± 0.0	108.3 ± 11.1	108.33 ± 11.34	261.8 ± 28.7	11.9 ± 1.7	425.00 ± 49.58	180.5 ± 18.0	77.3 ± 8.8
		Nrk	0.6 ± 0.4	114.3 ± 5.9	114.67 ± 6.46	251.5 ± 20.0	17.3 ± 1.1	382.67 ± 23.12	196.5 ± 21.8	95.5 ± 7.5
	Statistics (*F* value)									
	Prv		3.24*	7.42***	7.56***	4.62**	3.05*	4.53*	4.71**	3.87*
	CG		57.04***	63.71***	62.90***	12.08***	87.07***	7.8**	20.59***	80.16***
	Prv × CG		3.32*	3.31*	3.33*	1.2^ns^	1.07^ns^	1.03^n^	1.25^ns^	2.44*
Values represent means ± SE from three to five trees. Carotenoid of path A is *α*-carotene + lutein, and path B is the sum of *β*-carotene, Neo, xanthophyll cycle (VAZ), and Rho. Significant effects of Prv, CG, and their interaction are indicated as ^m^ *p* < 0.10, * *p* < 0.05, ** *p* < 0.01, *** *p* < 0.001, ^ns^ not significant.										

**Figure 3:**
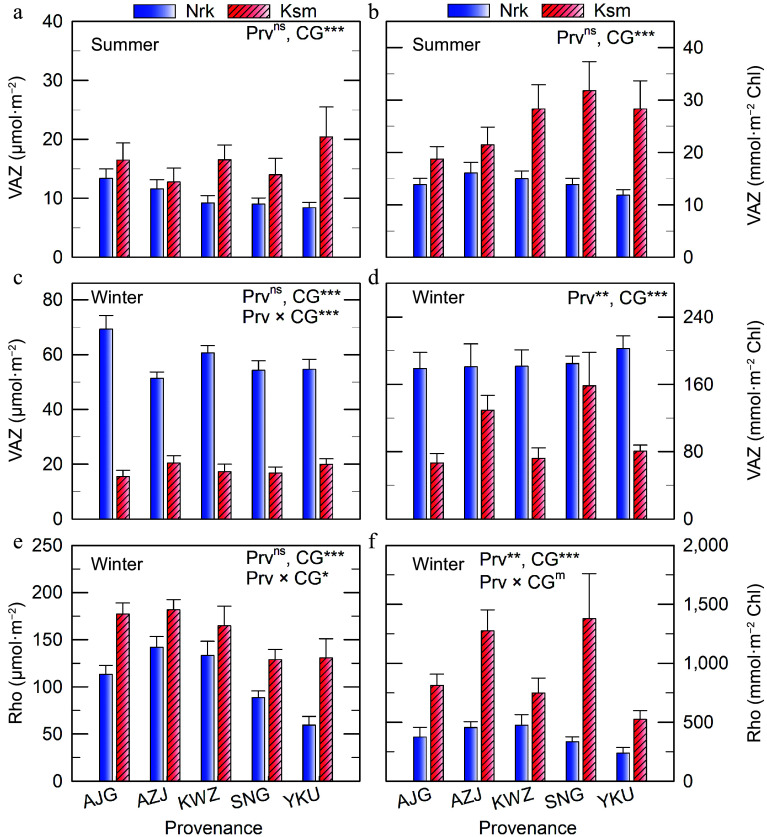
VAZ and Rho concentrations in needles of *C. japonica* from five Prv grown in two CG located in Nrk and Ksm. Values represent means ± SE from three to five trees, shown on a (a), (c), (e) projected area basis, and a (b), (d), (f) chlorophyll basis. Prv abbreviations are listed in Table 1. Significant effects of Prv, CG, and their interaction are denoted as ^m^ *p* < 0.10, * *p* < 0.05, ** *p* < 0.01, *** *p* < 0.001, ^ns^ not significant.

In summer, plants from all Prv exhibited similar VAZ concentrations, with lower levels observed in the colder CG on both area and chlorophyll bases (Fig. 3a, b). However, in winter, VAZ levels were higher in the colder CG (Fig. 3c, d), increasing 4.4–6.5-fold compared to summer when expressed per unit area. On a chlorophyll basis, the increase was even more pronounced.

Thermal dissipation capacity, as indicated by the proportion of A and Z in the VAZ pool, was lower in summer in the colder CG (Supplementary Fig. S3a). At night, over 90% of A and Z were epoxidized to V in both CGs (Supplementary Fig. S3b). In winter, A and Z were largely retained overnight in all Prv and CGs (Supplementary Fig. S3c, d).

Contrary to expectations, Rho concentrations were higher in the warmer CG (Fig. 3e). On a chlorophyll basis, Rho varied significantly by Prv, with wider variation observed in the warmer CG (Fig. 3f). Rho levels were 1.2–2.8 and 6.3–14.6 times higher than VAZ in the colder and warmer CGs, respectively.

The effects of Prv and CG on other carotenoids were season-dependent. In summer, Prv affected lutein, *α*-Car, and *β*-Car concentration, whereas the effect of CG was found on Neo and *α*-Car (Table 2). In winter, both factors significantly affected all carotenoids: levels were generally higher in the colder CG and in the northernmost AJG Prv. Notably, *α*-Car was downregulated in winter in both CGs of all Prv, absent in all but AJG in the warmer CG; lutein was upregulated in winter; Neo decreased in winter than in summer.

### Photochemical efficiency of photosystem II

**Figure 4:**
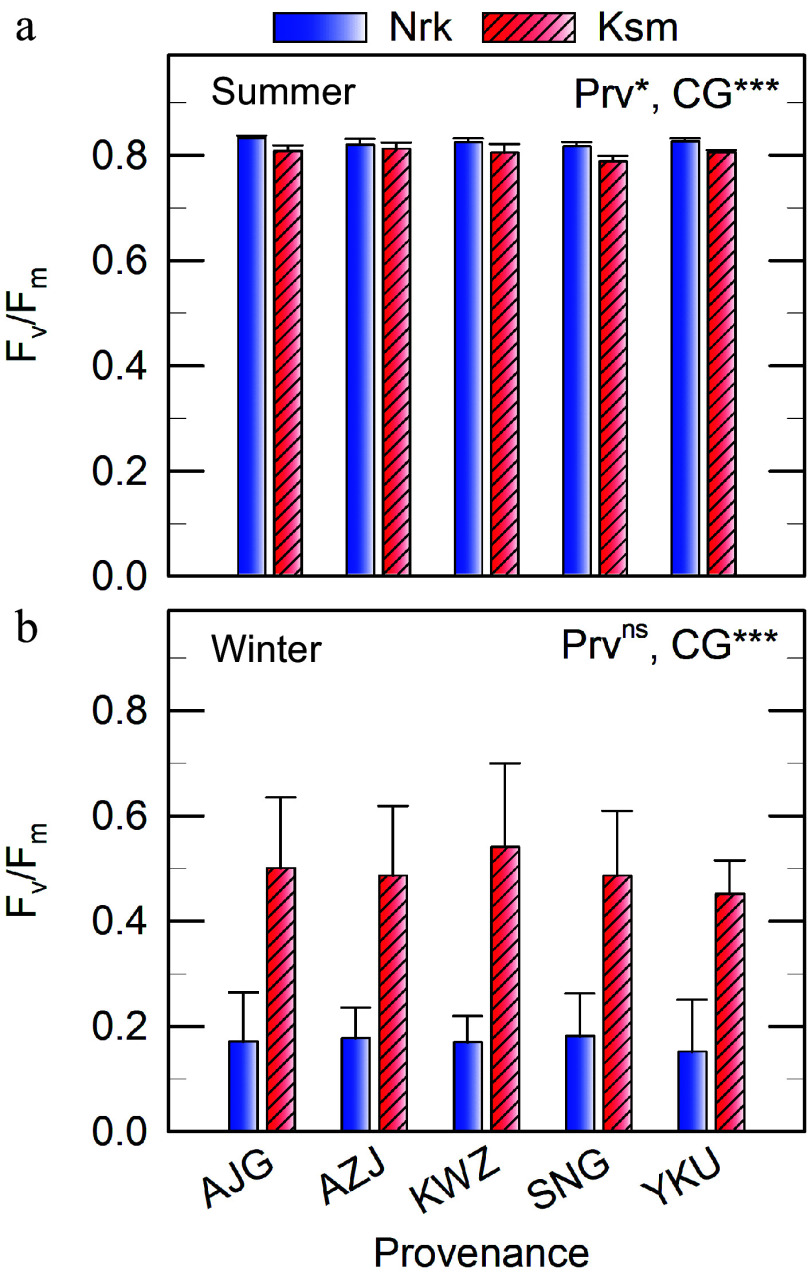
Dark-adapted photochemical efficiency of photosystem II (F_v_/F_m_) measured *in situ* 2 h after sunset on clear days in (a) summer and (b) winter in needles of *C. japonica* from five Prv grown in two CGs located in Nrk and Ksm. Values represent means ± SE from three to five trees. Prv abbreviations are listed in Table 1. Significant effects of Prv and CG are indicated as * *p* < 0.05, ** *p* < 0.01, *** *p* < 0.001, ^ns^ not significant.

In summer, F_v_/F_m_ values ranged from 0.79–0.83 across all Prv and CGs (Fig. 4a), suggesting the absence of chronic stress. Surprisingly, F_v_/F_m_ was slightly higher in the colder CG. In winter, F_v_/F_m_ declined in both CGs, but remained higher in the warmer CG (0.45–0.54) compared to the colder CG (0.15–0.18), with no significant effect of Prv (Fig. 4b).

### Photosynthetic parameters

A_max_ was similar across Prv and CGs in both seasons (Table 3), though it declined by about half in winter. In contrast, Ø varied by CG in summer, with the northernmost AJG exhibiting the highest value. Compared to its summer values, the decrease rate in both Ø and A_max_ in winter in the warmer CG was marginally lower than in the colder CG (*p* < 0.10). R_d_ was higher in the warmer CG, suggesting greater metabolic activity.

**Table 3:** Photosynthetic parameters in needles of *C. japonica* originated from three Prv growing in two CG and two-way ANOVA results.

Season	Prv	CG	Ø	*θ*	A_max_	R_d_
					μmol·m^−2^·s^−1^	
Summer	Ajigasawa (AJG)	Nrk	0.083 ± 0.010	0.86 ± 0.01	21.10 ± 1.60	2.38 ± 0.09
		Ksm	0.050 ± 0.005	0.68 ± 0.05	20.33 ± 2.10	2.93 ± 0.36
	Azouji (AZJ)	Nrk	0.063 ± 0.007	0.82 ± 0.04	20.89 ± 1.65	2.00 ± 0.70
		Ksm	0.060 ± 0.011	0.82 ± 0.05	17.31 ± 1.97	2.23 ± 0.25
	Singu (SNG)	Nrk	0.065 ± 0.008	0.82 ± 0.06	16.85 ± 0.59	1.73 ± 0.47
		Ksm	0.042 ± 0.004	0.73 ± 0.04	17.84 ± 0.59	1.97 ± 0.19
	Statistics					
	Prv		*p* = 0.308	*p* = 0.442	*p* = 0.134	*p* = 0.156
	CG		***p* = 0.010**	***p* = 0.022**	*p* = 0.391	*p* = 0.324
Winter	Ajigasawa (AJG)	Nrk	0.019 ± 0.005	0.76 ± 0.12	9.33 ± 2.35	2.73 ± 0.33
		Ksm	0.022 ± 0.006	0.45 ± 0.17	11.13 ± 2.47	3.97 ± 0.79
	Azouji (AZJ)	Nrk	0.023 ± 0.005	0.57 ± 0.16	10.84 ± 0.84	3.13 ± 0.57
		Ksm	0.024 ± 0.000	0.81 ± 0.00	12.14 ± 1.27	4.32 ± 0.09
	Singu (SNG)	Nrk	0.017 ± 0.003	0.30 ± 0.12	6.93 ± 1.17	3.06 ± 0.30
		Ksm	0.027 ± 0.009	0.03 ± 0.01	9.92 ± 2.01	3.65 ± 0.49
	Statistics					
	Prv		*p* = 0.813	*p* = 0.212	*p* = 0.268	*p* = 0.677
	CG		*p* = 0.267	*p* = 0.670	*p* = 0.192	***p* = 0.025**
Values shown are means ± SE from three trees of each CG. Ø: apparent quantum yield of CO_2_ fixation, *θ*: the convexity of the light-response curve, A_max_: the light-saturated photosynthetic rate, R_d_: dark respiration rate. Significant values are highlighted in bold.						

### Phenotypic plasticity

PIv was highest for pigments (0.334–0.455), followed by LMA (0.234–0.287), and lowest for F_v_/F_m_ (0.019–0.240) (Supplementary Fig. S4). Seasonal variation was evident only in PIv of F_v_/F_m_, which was higher in winter.

At the Prv level, PIv of pigments varied significantly among Prv and CGs (Supplementary Fig. S5). Within-Prv variation in pigment, PIv was comparable to that observed across Prv (Supplementary Figs S4 & S5).

### PCA analysis

**Figure 5:**
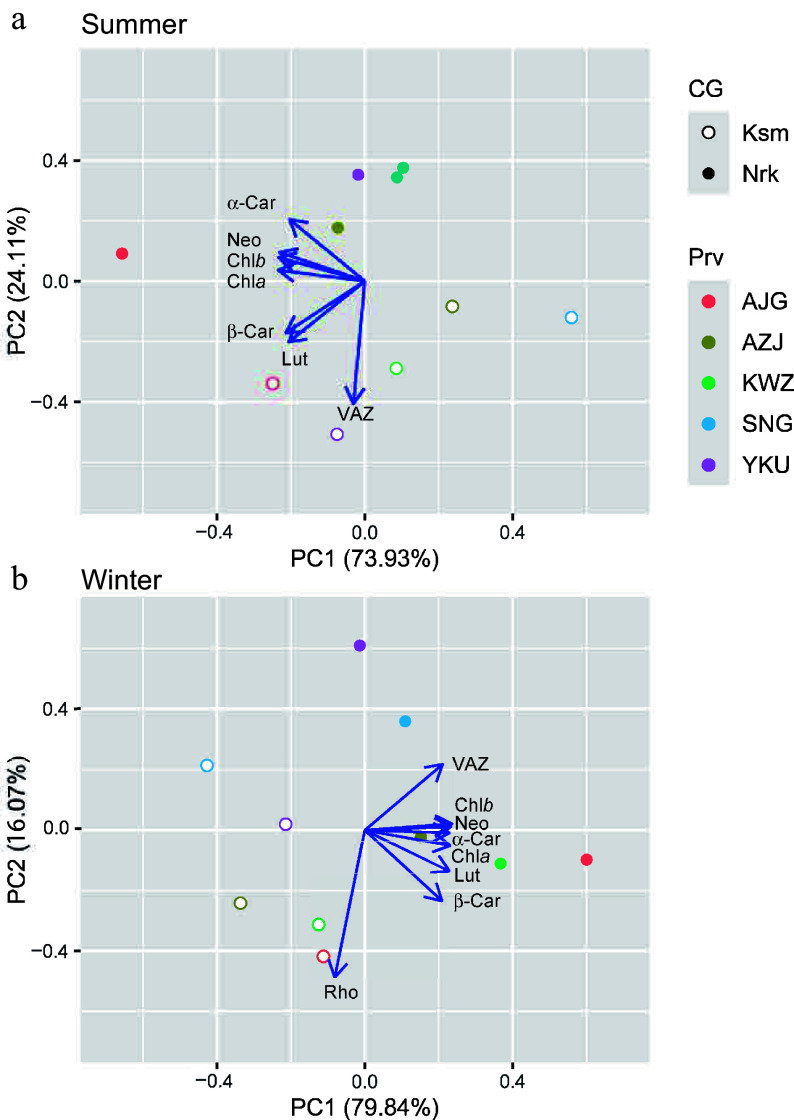
Principal component ordination of pigment components in needles of *C. japonica* from five Prv grown in two CG located in Nrk and Ksm in (a) summer and (b) winter. Prv abbreviations are listed in Table 1. Chl*a*: chlorophyll *a*, Chl*b*: chlorophyll *b*, VAZ: xanthophyll cycle, Rho: rhodoxanthin, Neo: neoxanthin, Lut: lutein, *α*-Car: *α*-carotene, *β*-Car: *β*-carotene.

In summer, PC1 and PC2 explained 73.93% and 24.11% of variance, corresponding to light absorption and thermal dissipation capacity, respectively (Fig. 5a). SNG from the 'Omote-sugi' in the warmer CG and the northernmost AJG in the colder CG were at opposite ends of PC1. Two CGs, regardless of their origin, were clearly distinct along the PC2. In winter, PC1 and PC2 explained 79.84% and 16.07% of the variance, representing light absorption/Rho accumulation and contrasting photoprotective strategies (VAZ vs Rho), respectively (Fig. 5b). Two CGs, regardless of their origin, were distinct along the PC1.

## Discussion

All *C. japonica* trees from the five Prv exhibited winter Chl reduction accompanied by upregulation of the VAZ cycle pool and Rho accumulation in both CGs. These seasonal pigment changes align with previous findings^[1,3,19]^, indicating that evergreen conifers employ pigment-mediated strategies to endure winter stress. Notably, significant genetic variation was observed in the degree of Chl reduction across Prv and CGs. Unexpectedly, higher Chl concentrations in winter were found in the colder CG, especially in the northernmost Prv. The risk of high excitation pressure in PSII was mitigated by a six-fold increase in VAZ (sustained AZ) and elevated lutein levels—both recognized mechanisms of cold acclimation^[1,3]^. Conversely, Rho levels were lower in the colder CG, contrary to findings along elevational gradients from the same species^[24]^. This suggests that winter severity regulates the relative deployment of VAZ- and Rho-mediated photoprotection, with genotype-dependent plasticity. Despite variations in pigment composition, F_v_/F_m_ remained comparable among Prv, consistently higher in the warmer CG, while photosynthetic parameters showed slight variation. Together with greater phenotypic plasticity in pigment traits and LMA at inter- and intra-Prv levels, these results imply that *C. japonica* possesses adaptive resilience to climatic changes, as seen in its robust performance under high summer temperatures in the warmer CG.

### Integrated variation in pigment components

Seasonal Chl reduction is a hallmark of conifer cold acclimation, yet species-specific patterns and mechanisms remain elusive^[4,11]^. These results suggest that pigment variation reflects a strategic balance between maximizing photosynthesis and minimizing stress, shaped by environmental context. In the colder CG, high Chl and coordinated carotenoids indicate an adaptive strategy for a short growing season. High Chl*b* in summer correlated with enhanced quantum yield of CO_2_ fixation in the northernmost Prv. The presence of substantial *α*-Car in summer, followed by marked depletion in winter, underscores this strategy because it is often upregulated under low light^[39]^. Winter upregulation of lutein, synthesized from *α*-carotene, led to an increased lutein : Chl ratio—a known response to cold stress^[9]^—and supports its role in quenching chlorophyll triplet states^[42]^. Moreover, *β*-Car levels, localized exclusively in PSI and PSII core complexes^[9]^, were maintained in winter and higher in the northernmost Prv, implying that winter Chl reduction targets the antenna while core complexes remain as observed in *Pinus sylvestris*^[43]^. This structural reconfiguration may facilitate energy transfer from PSII to PSI^[1]^. Enhanced VAZ and sustained AZ level provided additional photoprotection via energy dissipation. Furthermore, seasonal reduction in Neo—exclusively associated with the PSII antenna and synthesized from zeaxanthin^[44]^—supports this interpretation. These results suggest that coordinated Chl and carotenoid adjustments, along with fluxes through *α*- and *β*-carotene pathways, regulate the pools of lutein, VAZ cycle, and Rho^[39]^.

Such integration may enhance photosynthetic recovery in spring. Chl resynthesis is energetically costly^[45]^, while conversion of sustained Z back to V occurs within days^[3,46,47]^, enabling rapid functional shifts in PSII from photoprotection to light harvesting^[1,3]^. These dynamics support earlier observations linking pigment plasticity with spring productivity increases in high-latitude conifers under global warming^[48,49]^. Given the limited data on interspecific variation in photosynthetic recovery in spring^[50−53]^, further research is needed to clarify species-specific responses and their broader implications for forest carbon dynamics^[54,55]^.

Although cold winters are typically considered the primary selective force in *C. janopica*^[33]^, these findings also highlight the importance of hot summers. PCA of climatic data identified the Ksm CG as having the hottest summer and greatest precipitation seasonality. The SNG Prv, from the Pacific-side 'Omote-sugi' group, showed low Chl and high VAZ when grown in the Ksm CG, consistent with adaptation to drought and heat stress in root and growth traits^[31,56]^. Furthermore, higher VAZ pool per Chl in the Ksm CG supports its role in photoprotection under summer stress. These trends were corroborated by PCA of pigment composition. Both genetic diversity and phenotypic plasticity in pigments and LMA appear essential for maintaining photosynthetic function across variable environments.

### Photoprotective role of carotenoids

Contrary to expectations, Rho accumulation was greater in the warmer CG. The balance between VAZ and Rho may depend on winter severity and the rate of pigment synthesis and degradation. Rho synthesis, likely triggered by sustained Z^[22]^, proceeds more slowly than the VAZ cycle^[14,19,24]^. Notably, experimental Rho degradation required 10 d at room temperature^[24]^, considerably longer than Z-epoxidation^[3,39]^. In *C. japonica*, F_v_/F_m_ did not fully recover even after degradation of Rho when it grew under the minimum temperature below –10 °C^[24]^, indicating a decoupling of pigment dynamics and PSII recovery under harsher conditions. This aligns with the natural distribution of Rho-accumulating conifers, which typically inhabit milder winter regions.

These findings support Rho's role in light interception under moderate winter stress^[19]^. In winter, higher Rho and lower VAZ in the warmer CG coincided with smaller decreases in quantum yield of CO_2_ fixation and A_max_. Rho accumulation is associated with chloroplast-to-chromoplast conversion in sun-exposed tissues^[21,25]^, restricting PSII excitation under low light while allowing limited photosynthetic activity when irradiance increases^[19]^.

## Conclusions

Understanding how *C. japonica* regulates chlorophyll and carotenoids across thermal gradients is critical for assessing resilience to climate change. These findings reveal that higher winter chlorophyll, particularly in colder sites, reflects adaptation to short growing seasons. Photoprotective strategies—via VAZ or Rho—varied across sites and Prv, but pigment plasticity ensured consistent photochemical efficiency. Integrating remote sensing indices sensitive to foliar chlorophyll and carotenoid dynamics^[55,57,58]^, may improve carbon cycle estimates under climate change scenarios.

## SUPPLEMENTARY DATA

Supplementary data to this article can be found online.

## Data Availability

The datasets generated during and/or analyzed during the current study are available from the corresponding author on reasonable request.
