# Supplementary material for: Photoprotective pigment plasticity and cold acclimation strategies in Cryptomeria japonica across two common gardens
Source: For Res (Fayettev). 2025 Jul 31;5:e015. doi: 10.48130/forres-0025-0015 (PMC12441905; doi:10.48130/forres-0025-0015)
Supplement: Supplementary file 1 — Supplementary data to this article can be found online. [file FR-2025-5-0015-Supplementary.zip › 10.48130_forres-0025-0015-Suppl-FigureS3.pdf]

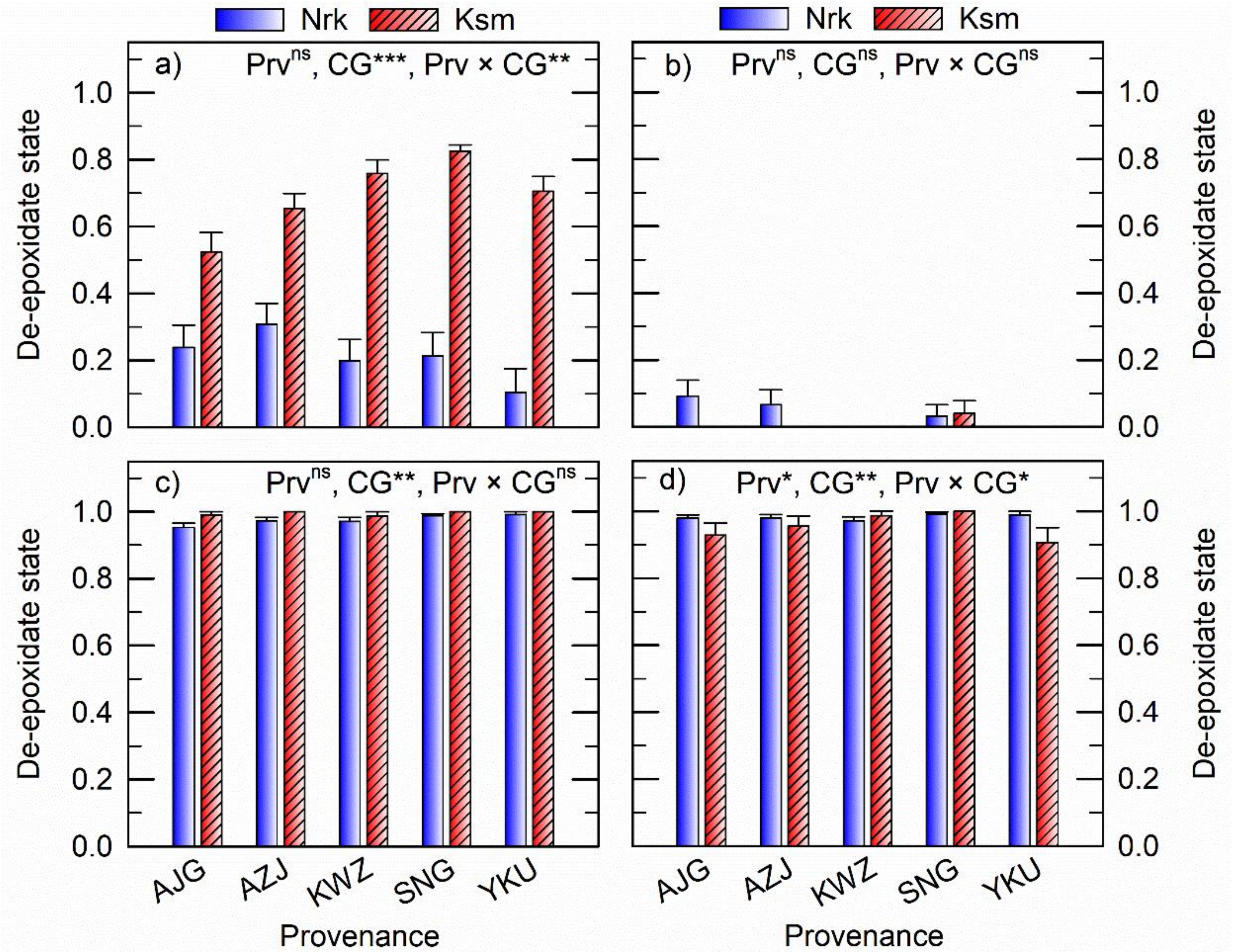

Fig. S3. De-epoxidation state (AZ/VAZ ratio) in needles of *Cryptomeria japonica* from five provenances (Prv) grown in two common gardens (CG) located in Miyagi (Nrk) and Kumamoto (Ksm), sampled at (a, c) noon and (b, d) night in (a, b) summer and (c, d) winter. Values represent means ± SE from 3-5 trees. Provenance abbreviations are listed in Table 1. Significant effect of Prv and CG are indicated as * $P < 0.05$, ** $P < 0.01$, *** $P < 0.001$, ^{ns} not significant. V: violaxanthin; A: antheraxanthin, Z: zeaxanthin.
