# Supplementary material for: Photoprotective pigment plasticity and cold acclimation strategies in Cryptomeria japonica across two common gardens
Source: For Res (Fayettev). 2025 Jul 31;5:e015. doi: 10.48130/forres-0025-0015 (PMC12441905; doi:10.48130/forres-0025-0015)
Supplement: Supplementary file 1 — Supplementary data to this article can be found online. [file FR-2025-5-0015-Supplementary.zip › 10.48130_forres-0025-0015-Suppl-FigureS4.pdf]

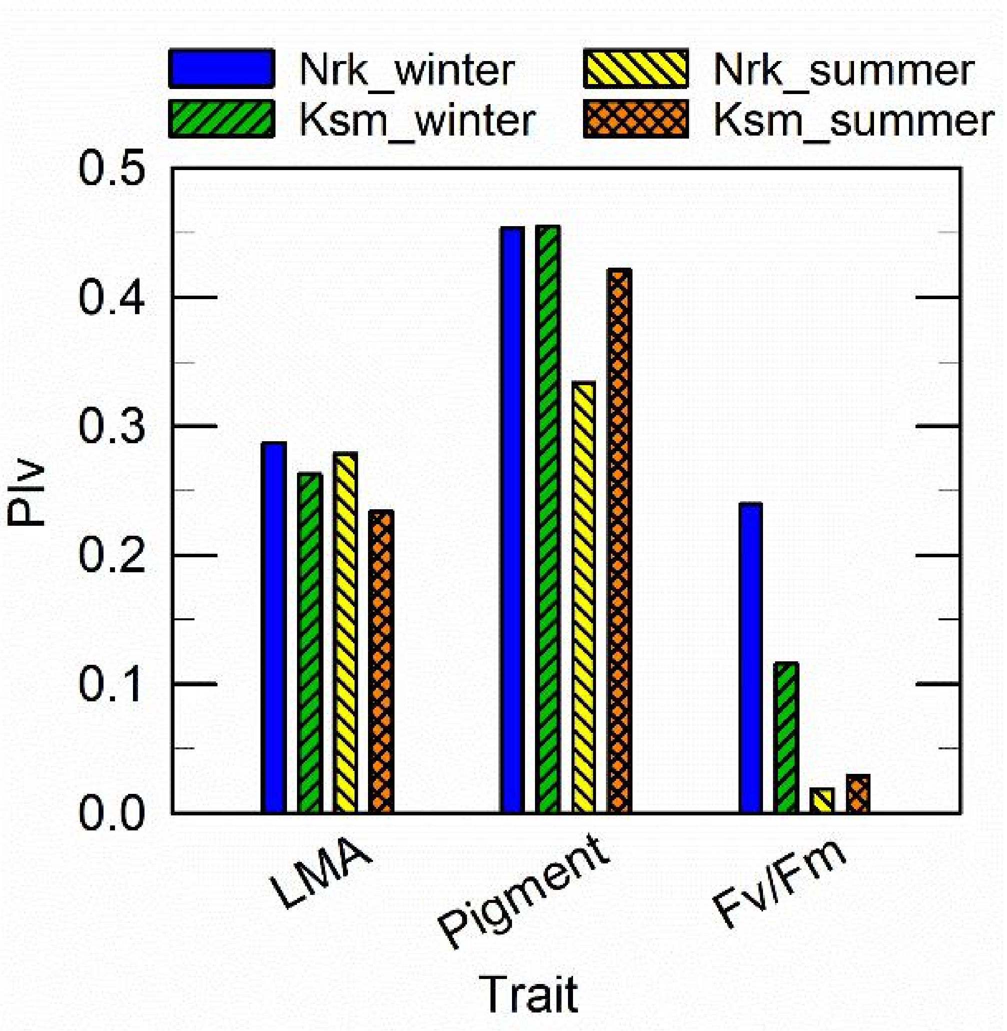

Figure S4 Comparison of phenotypic plasticity index (PIv) in morphological (LAM: leaf mass per area) and physiological traits in needles of *Cryptomeria japonica*, averaged across five provenances grown in two common gardens located in Miyagi (Nrk) and Kumamoto (Ksm). Pigment PIv values represent the average of all components, including chlorophyll *a*, chlorophyll *b*, xanthophyll cycle, neoxanthin, lutein, α-carotene and β-carotene in summer, and surplus rhodoxanthin in winter.
