# Supplementary material for: Photoprotective pigment plasticity and cold acclimation strategies in Cryptomeria japonica across two common gardens
Source: For Res (Fayettev). 2025 Jul 31;5:e015. doi: 10.48130/forres-0025-0015 (PMC12441905; doi:10.48130/forres-0025-0015)
Supplement: Supplementary file 1 — Supplementary data to this article can be found online. [file FR-2025-5-0015-Supplementary.zip › 10.48130_forres-0025-0015-Suppl-FigureS5.pdf]

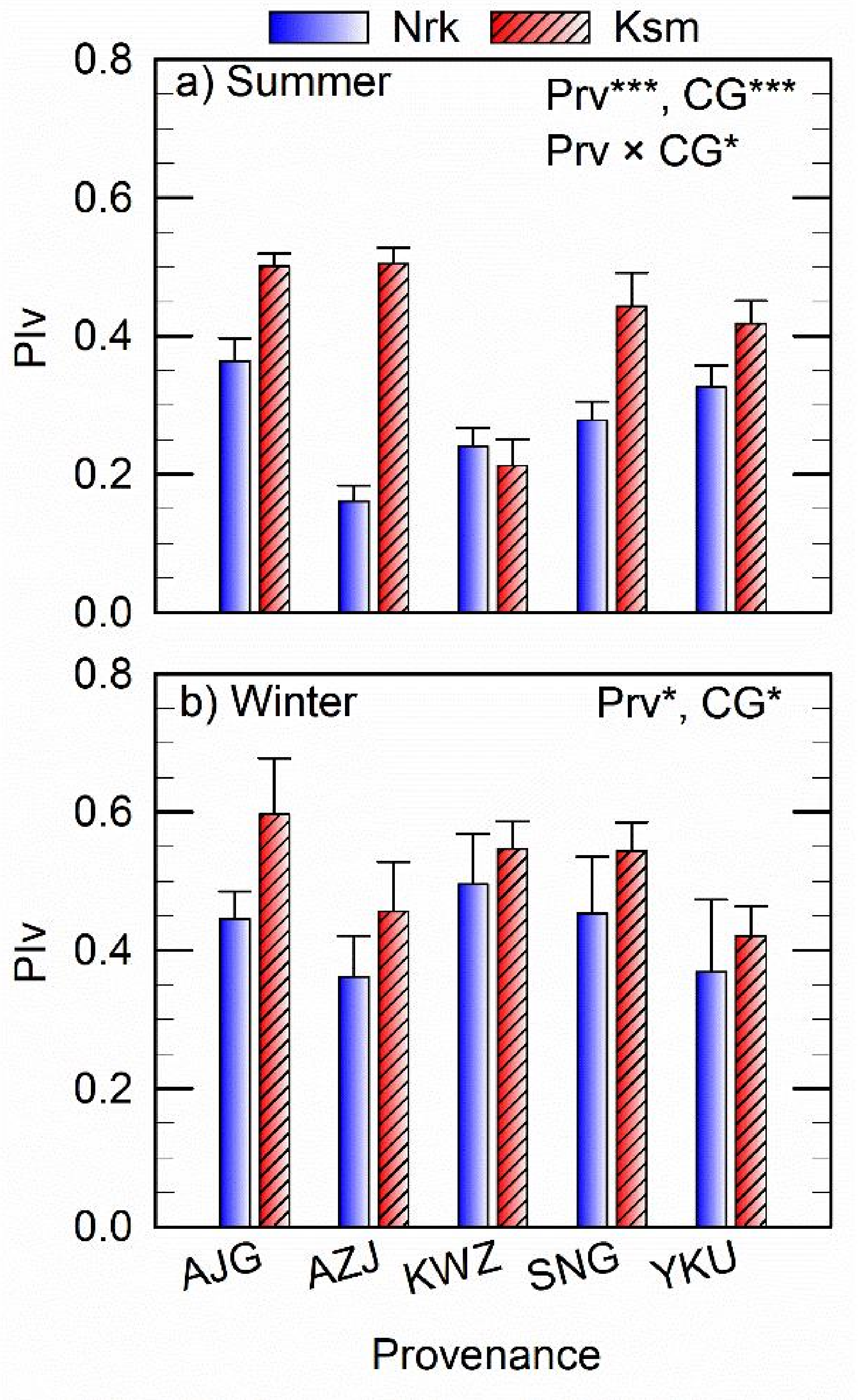

Fig. S5 Mean phenotypic plasticity index (PIv) of pigments in each provenance of *Cryptomeria japonica* from five provenances (Prv) grown in two common gardens (CG) located in Miyagi (Nrk) and Kumamoto (Ksm). Values represent means ± SE of each pigment component in either summer or winter. Provenance abbreviations are listed in Table 1. Significant effect of Prv and CG are indicated as * $P < 0.05$, *** $P < 0.001$.
