# Supplementary material for: Photoprotective pigment plasticity and cold acclimation strategies in Cryptomeria japonica across two common gardens
Source: For Res (Fayettev). 2025 Jul 31;5:e015. doi: 10.48130/forres-0025-0015 (PMC12441905; doi:10.48130/forres-0025-0015)
Supplement: Supplementary file 1 — Supplementary data to this article can be found online. [file FR-2025-5-0015-Supplementary.zip › 10.48130_forres-0025-0015-Suppl-TableS1.pdf]

Table S1. F values from ANOVA based on linear mixed-effects models for total chlorophyll (Chl) and each carotenoid components per unit needle dry weight in *Cryptomeria japonica* from five provenances (Prv) cultivated in two common gardens (CG).

Season	Source of variation	numDF	Chl	VAZ	Rho	Neo	Lut	α-Car	β-Car
Summer	Prv	4	0.95^{ns}	0.24^{ns}	—	1.86^{ns}	1.64^{ns}	0.91^{ns}	2.14^{ns}
	CG	1	36.17^{***}	10.40^{**}	—	58.73^{***}	0.13^{ns}	93.65^{***}	2.39^{ns}
	Prv×CG	4	1.78^{ns}	1.02^{ns}	—	2.94^{*}	1.37^{ns}	0.70^{ns}	0.67^{ns}
Winter	Prv	4	2.92^{*}	2.88^{m}	1.57^{ns}	1.58^{ns}	4.19^{*}	1.86^{ns}	2.73^{*}
	CG	1	39.74^{***}	634.34^{***}	63.84^{***}	106.46^{***}	83.01^{***}	58.73^{***}	16.94^{***}
	Prv×CG	4	0.34^{ns}	5.71^{***}	2.81^{**}	1.16^{ns}	3.17^{*}	2.94^{*}	1.20^{ns}

VAZ: xanthophyll cycle, Rho: rhodoxanthin, Neo: neoxanthin, Lut: lutein, α-Car: α-carotene, β-Car: β-carotene. * $P < 0.05$, ** $P < 0.01$, *** $P < 0.001$, ^{ns} not significant
