# Supplementary material for: Photoprotective pigment plasticity and cold acclimation strategies in Cryptomeria japonica across two common gardens
Source: For Res (Fayettev). 2025 Jul 31;5:e015. doi: 10.48130/forres-0025-0015 (PMC12441905; doi:10.48130/forres-0025-0015)
Supplement: Supplementary file 1 — Supplementary data to this article can be found online. [file FR-2025-5-0015-Supplementary.zip › 10.48130_forres-0025-0015-Suppl-TableS2.pdf]

Table S2. Chlorophyll (Chl) *a*:*b* ratio and molar ratios of individual carotenoid components to total Chl (mol mol^{-1}) in needles of *Cryptomeria japonica* from five provenances (Prv) grown in two common gardens (CG), along with statistical summary.

Season	Provenance	CG	Chl *a*:*b*	Lut/Chl	β-Car/Chl*a*	Total Car/Chl
Summer	AJG	Ksm	3.32±0.12	0.18±0.01	0.66±0.05	0.76±0.04
		Nrk	3.11±0.14	0.15±0.01	0.57±0.04	0.67±0.03
	AZJ	Ksm	2.90±0.15	0.20±0.01	0.84±0.07	0.91±0.06
		Nrk	3.20±0.12	0.15±0.01	0.58±0.03	0.68±0.02
	KWZ	Ksm	3.11±0.12	0.19±0.01	0.70±0.04	0.81±0.03
		Nrk	2.96±0.13	0.15±0.01	0.60±0.03	0.69±0.03
	SNG	Ksm	3.09±0.12	0.20±0.01	0.79±0.04	0.89±0.03
		Nrk	3.18±0.08	0.14±0.00	0.52±0.02	0.63±0.02
	YKU	Ksm	3.31±0.13	0.18±0.01	0.66±0.03	0.78±0.02
		Nrk	3.20±0.07	0.14±0.00	0.56±0.02	0.66±0.02
	Statistics (*F* value)					
	Prv		0.20^{ns}	0.33^{ns}	0.40^{ns}	0.38^{ns}
	CG		0.20^{ns}	69.27^{***}	30.22^{***}	46.31^{***}
	Prv×CG		2.00^{ns}	1.77^{ns}	2.62^{*}	2.65^{*}
Winter	AJG	Ksm	2.32±0.17	0.58±0.06	2.07±0.28	2.89±0.28
		Nrk	1.95±0.12	0.51±0.06	1.56±0.24	2.11±0.25
	AZJ	Ksm	2.25±0.13	0.68±0.06	2.46±0.24	3.80±0.38
		Nrk	2.00±0.10	0.45±0.04	1.47±0.16	2.13±0.21
	KWZ	Ksm	2.32±0.14	0.50±0.05	1.72±0.17	2.55±0.27
		Nrk	2.05±0.09	0.45±0.04	1.53±0.12	2.20±0.22
	SNG	Ksm	2.64±0.16	0.71±0.12	2.19±0.32	3.88±0.78
		Nrk	1.75±0.08	0.45±0.02	1.54±0.11	2.01±0.11
	YKU	Ksm	2.40±0.12	0.43±0.03	1.49±0.12	2.12±0.16
		Nrk	2.03±0.09	0.42±0.03	1.36±0.09	1.82±0.11
	Statistics (*F* value)					
	Prv		0.19^{n}	3.30^{*}	1.79^{ns}	3.80^{*}
	CG		45.24^{***}	4.08^{*}	5.56^{*}	10.74^{**}
	Prv×CG		3.06^{*}	1.39^{ns}	1.21^{ns}	1.72^{ns}

Total carotenoid (Total Car) includes neoxanthin, lutein (Lut), α-carotene, β-carotene (β-Car), xanthophyll cycle pigments (vioxanthin, antheraxanthin, and zeaxanthin) in summer, and surplus rhodoxanthin in winter. * $P < 0.05$, ** $P < 0.01$, *** $P < 0.001$, ^{ns} not significant
