# Supplementary material for: Photoprotective pigment plasticity and cold acclimation strategies in Cryptomeria japonica across two common gardens
Source: For Res (Fayettev). 2025 Jul 31;5:e015. doi: 10.48130/forres-0025-0015 (PMC12441905; doi:10.48130/forres-0025-0015)
Supplement: Supplementary file 1 — Supplementary data to this article can be found online. [file FR-2025-5-0015-Supplementary.zip › 10.48130_forres-0025-0015-Suppl-FigureS1.pdf]

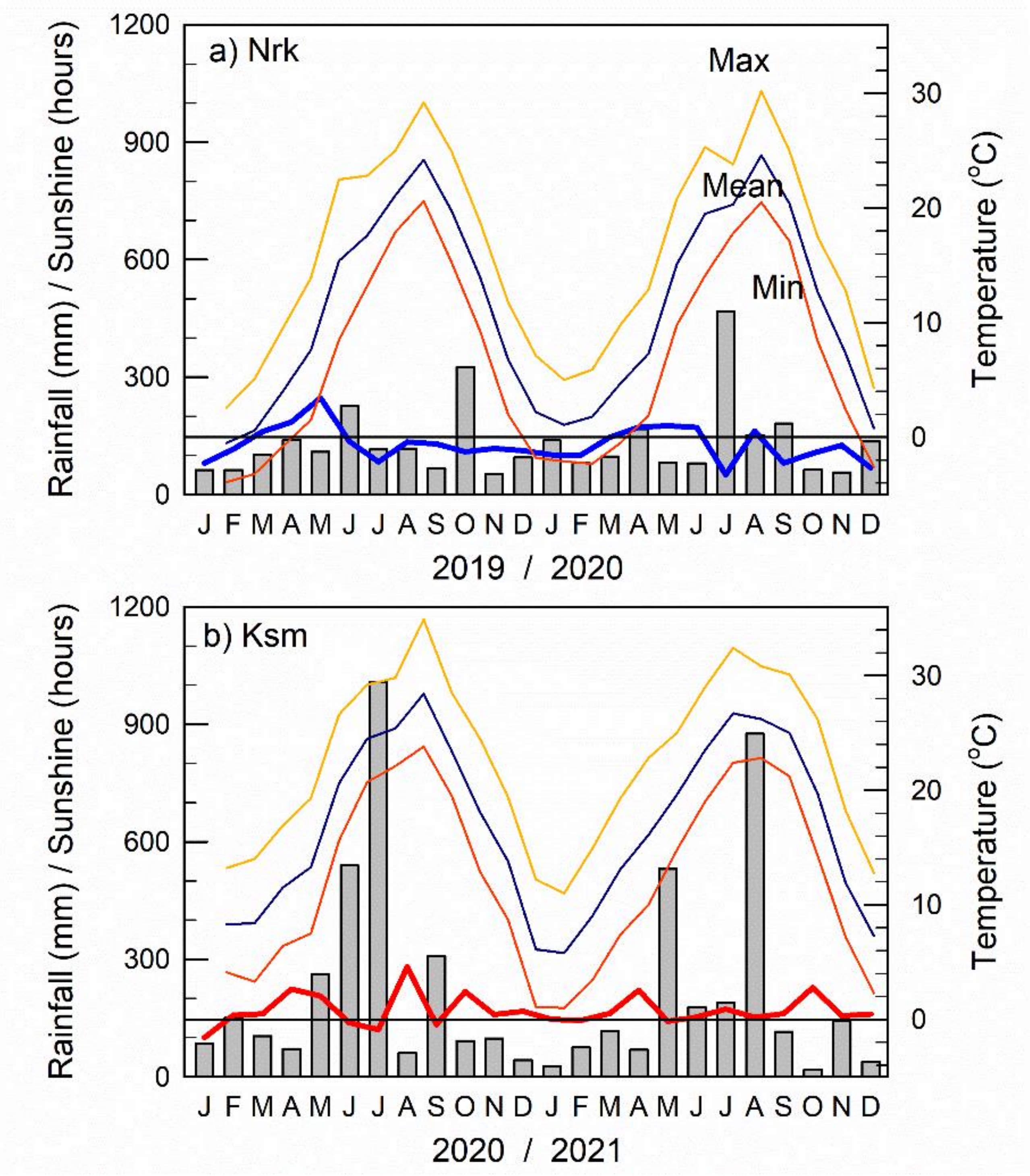

Fig. S1. Monthly rainfall (bars, left axis) and sunshine hours (thick line, left axis), and mean daily mean temperature, daily minimum and maximum temperature (thin lines, right axis) in the year when leaves were sampled from the common garden of Miyagi Prefecture (Nrk) in 2019-2020 and Kumamoto Prefecture (Ksm) in 2020-2021. Data from Japan Meteorological Agency (https://www.data.jma.go.jp/stats/etrn/index.php).
